# Glial-neuronal ensembles: partners in drug addiction-associated synaptic plasticity

**DOI:** 10.3389/fphar.2014.00204

**Published:** 2014-09-02

**Authors:** Jean Lud Cadet, Veronica Bisagno

**Affiliations:** ^1^Molecular Neuropsychiatry Research Branch, Intramural Research Program, National Institute on Drug Abuse-NIHBaltimore, MD, USA; ^2^Instituto de Investigaciones Farmacológicas (ININFA-UBA-CONICET)Buenos Aires, Argentina

**Keywords:** synaptic plasticity, drug abuse, microglia, astroglia, oligodendroglia, inflammation

## Introduction

Drug addiction is manifested by a compulsive drive to take licit or illicit substances despite repeated severe adverse consequences (Volkow et al., 2012). Addiction is also accompanied by a vicious cycle of binges, abstinence, and relapses. Almost all drugs of abuse trigger euphoric feelings consequent to a rapid increase of dopamine levels in the mesolimbic system. Even after long periods of abstinence, addicts remain vulnerable to drug craving and/or relapses that can be triggered by stimuli previously associated with drugs (Koob and Volkow, 2010). These features of addiction suggest that drugs might cause a form of persistent neuroplasticity that is acutely responsive to environmental stimuli, with consequent compulsive drug-seeking and taking behaviors.

Neural functions require the coordinated interactions of multiple neuronal cell types and a diverse population of glial cells. The three major glial cell types in the brain, astrocytes, oligodendrocytes, and microglia, communicate with each other and with neurons by using neurotransmitters, other small molecules, and gap junctions (Araque et al., 2014). Oligodendrocytes increase the speed of electrical transmission through nerve axons by forming the axonal myelin sheath and clustering ion channels at nodes of Ranvier (Nave, 2010). Microglia prune synapses in part by monitoring synaptic transmission (Schafer et al., 2013; Wake et al., 2013). Astrocytes can regulate synaptic transmission between neurons by modifying the concentration of extracellular potassium, controlling local blood flow, by releasing and/or taking up neurotransmitters or neuromodulators, by delivering nutrients to neurons, and by altering the geometry and volume of the brain extracellular space (Araque et al., 2014).

This brief summary of glial functions suggests that these cells might play important roles in the long-term manifestations of substance use disorders, both in terms of addiction to these agents and their long-term neuropsychiatric consequences. In what follows, we discuss some recent findings that support the thesis that glial cells are part and parcel of the plastic mechanisms that are induced by drugs of abuse.

### Brain inflammation triggered by drugs of abuse

Gliosis and inflammatory responses are significant pathological features of substance use disorders (Cadet et al., 2014a). Inflammation is a natural response to damage and/or infection that are mediated by pro-inflammatory cytokines including interleukin 1 beta (IL-1β), interleukin 6 (IL-6), and tumor necrosis factor alpha (TNFα) (Glass et al., 2010). In the brain, microglial cells are the main orchestrators of these neuroinflammatory responses (Jeong et al., 2013). However, other cells including astrocytes, endothelial cells, perivascular and meningeal macrophages, and even neurons, can also produce pro-inflammatory mediators (Van Wagoner et al., 1999; Jeong et al., 2013). These factors appear to mediate some detrimental effects of inflammation on neurogenesis (Sierra et al., 2014).

Brain inflammation is also associated with an increased production of reactive oxygen species (ROS) and nitric oxide (NO), followed by the propagation of free radicals that damage cells (Cadet and Brannock, 1998; Krasnova and Cadet, 2009). Indeed, various psychostimulants, including amphetamine and methamphetamine, can produce ROS in dopaminergic nerve terminal regions (Krasnova and Cadet, 2009; Shiba et al., 2011). Acute or repeated cocaine administration also generates ROS in dopaminergic rat brain structures (Dietrich et al., 2005). MDMA also produces reactive nitrogen species in the rat that contribute to its neurotoxicity (O'Shea et al., 2014).

### Drugs abuse-induced alterations in glial cells

#### Astroglia

Astrocytes play diverse roles in the regulation of synaptic transmission. They clear synaptic transmitters from the cleft through the activity of transporters and can recycle glutamate through a glutamine intermediate to the synaptic terminal (Haydon et al., 2009). Astrocytes can also release glutamate (D'Ascenzo et al., 2007; Jourdain et al., 2007), the NMDA receptor co-agonist D-serine (Mothet et al., 2005), and ATP (Cotrina et al., 2000). Hydrolysis of ATP to adenosine is responsible for an adenosine 1 (A1) receptor-mediated presynaptic inhibition of excitatory synaptic transmission (Volterra and Meldolesi, 2005).

GFAP-positive glial cells in the mPFC contain cystine/glutamate antiporters (Pow, 2001) that maintain extracellular non-synaptic glutamate levels and provide functional support to neurons by regulating extracellular potassium and the reuptake of glutamate at synapses (Wigley et al., 2007). Interestingly, non-synaptic glutamate derived from cystine/glutamate antiporters has been reported to modulate synaptic glutamate release and to regulate cocaine-induced drug seeking in rats (Moran et al., 2005). Moreover, down-regulation of the cystine/glutamate exchanger was reported to account for chronic cocaine-induced reduction in basal glutamate levels (Baker et al., 2003). Furthermore, the sodium-dependent glutamate uptake and the membrane level of the primary glial glutamate transporter (GLT1) were recently reported to be reduced in the nucleus accumbens upon withdrawal from self-administered cocaine (Scofield and Kalivas, 2014). It is important to note that Narita et al. (2006) have indicated that astrocyte-, but not microglia-, related soluble factors were able to amplify both methamphetamine- and morphine-dependent rewarding effects. Astrocytic control of glutamatergic signaling during abstinent periods may also critically impact reinstatement of drug-seeking behaviors (Turner et al., 2013). These conclusions stem from studies in which a glia-selective dominant-negative SNARE protein was expressed in mice and subsequently used to assess the contribution of glial transmission on cocaine-induced behaviors. The authors were able to demonstrate that glial transmission is necessary for reinstatement of drug-seeking behaviors triggered by cocaine or associated cues (Turner et al., 2013).

Although much remains to be done to clarify the role of astrocytes in drug-induced behaviors, some of their behavioral effects might be consequences to their production of trophic factors that can impact adult neurogenesis (Barkho et al., 2006). This idea is supported by the evidence that exposure to drugs of abuse can influence neurogenesis (Mandyam and Koob, 2012). It is important to also note that gliogenesis in the mPFC is altered after self-administration of various drugs of abuse (Mandyam and Koob, 2012). Taken together, the impact of drugs of abuse on both neurogenesis and gliogenesis might create an environment that is permissive to the generation and persistence of long-term memories associated with the addictive process.

It is also important to note that the glial-derived neurotrophic factor (*G*DNF) has also been studied in animal models of addiction (Yan et al., 2007, 2013; Lu et al., 2009). This factor provides trophic support to dopamine neurons and modulates midbrain microglial activation (Rocha et al., 2012). GDNF-dependent neuroadaptations in midbrain VTA neurons appear to play an important role in the development of incubation of cocaine craving (Lu et al., 2009). GDNF expression may be also associated with enduring vulnerability to reinstatement of METH-seeking behavior (Yan et al., 2007, 2013). More studies need to be conducted to elucidate how GDNF might influence dopaminergic functions in other brain regions after chronic exposure to psychostimulants and other drugs of abuse.

#### Oligodendroglia

Oligodendrocytes are cell types responsible for providing myelin for rapid propagation of action potentials (Nave, 2010). The brain contains an abundant class of progenitor cells that express the chondroitin sulfate proteoglycan, NG2, and the alpha receptor for platelet-derived growth factor (PDGFαR) (Nishiyama, 2007). These NG2^+^glial cells are called oligodendrocyte precursor cells (OPCs) because they generate oligodendrocytes during early postnatal development (Nishiyama, 2007). OPCs remain abundant in the adult CNS and retain the ability to differentiate into oligodendrocytes (Kang et al., 2010). They can regenerate oligodendrocytes after their degeneration through chemical- or autoimmune-mediated demyelination (Franklin and Ffrench-Constant, 2008). Oligodendrocytes can also regulate axonal function via their influence on neuron-glial interactions (Fields, 2014)

At present, there is very little information available on the potential effects of drugs of abuse on oligodendrocytes. For example, Lin et al. (2013) used a 3.0-Tesla MR scanner to study the brains of 34 heavy smokers and compared them to those of 34 age- and sex-matched controls. They found that heavy smokers had lower fractional anisotropy in the left anterior corpus callosum, an area that corresponded to the genu and rostral body of the corpus callosumm. These smokers did not show any area of increased anisotropy. They reported further that these smokers showed decreased axial diffusivity and increased radial diffusivity, but no changes in mean diffusivity. The authors suggested that their observations might the results of axonal loss and disrupted myelin integrity (Fields, 2014). Importantly, regression analysis revealed that these changes were related to the duration of smoking, thus suggesting that long-term exposure to nicotine and/or other factors in smoke might damage or impair the functions of oligodendrocytes. These observations are consistent with previous observations in chronic cigarette smokers (Paul et al., 2008). It is also important to mention that nicotine can cause significant increases in myelin genes in the prefrontal cortex, caudate putamen, and the nucleus accumbens of rats exposed to the drug *in utero* (Cao et al., 2013). The impact of these changes on neuronal functions will need to be investigated further.

Animal studies have also revealed certain white matter abnormalities after extended cocaine use. For example, George et al. (2008) investigated memory functions in rats that had 6-h access pre-day to cocaine. These rats escalated their intake of cocaine and exhibited working memory deficits. In addition, there was a significant correlation between decreased NG2-positive cells and cognitive impairments in these rats. Other investigators have also reported that chronic cocaine can cause decreased level of white matter proteins in the mouse nucleus accumbens (Kovalevich et al., 2012). Opioids also appear to affect the functions of oligodendrocytes. Specifically, perinatal exposure to buprenorphine has been shown to influence brain myelination, in that some doses of the buprenorphine were associated with reduced nuber of myelinatied axons (Sanchez et al., 2008). Buprenorphine also caused an inverted U-type increases in myelin basic proteins (MBPs), with the highest doses causing normalization of the levels of MBPs (Eschenroeder et al., 2012). Low doses of bupenorphine also increeased morphological complexity and increased the percentage of pre-oligodendrocytes that reach maturity. These differentiating effects appear to be mediated by stimulation of mu-opioid receptors (Eschenroeder et al., 2012). In contrast, higher does of the drug might exert their influence through the nociceptin/orphanin FQ (NOP) receptor (Eschenroeder et al., 2012). These observations suggest that further evaluation of oligodendryte functions in adults being treated with opioid agents are necessary.

These animal studies are consistent with the suggestion that myelin dysfunction might account for some of the deficits in white matter integrity described in studies of humans addicted to various substances (Cadet et al., 2014a). More studies are needed to elucidate if electrophysiological abnormalities observed in some models of addiction might be secondary to drug-induced myelin dysfunction and associated abnormalities in conduction of action potentials to synaptic areas.

#### Microglia

Microglial cells are the immune cells that reside in the brain parenchyma (Sierra et al., 2014). They are exceptional sensors of their microenvironment and respond by undergoing remarkable changes in morphology and gene expression (Aguzzi et al., 2013). During pathological insults, activated microglial cells thicken and retract their processes, extend filopodia, proliferate and migrate. They also release factors and compounds that can influence neuronal survival. These factors include proinflammatory cytokines, trophic factors, and ROS. They also phagocytose pathogens, degenerating cells, and debris (Schafer et al., 2013). Of relatedness to our present discussion, reactive microgliosis has been detected in several regions of the brains of methamphetamine addicts who had been abstinent for several years (Sekine et al., 2008). These results had suggested that methamphetamine exposure had engendered a process that had enduring effects on the proliferation of reactive microglial cells. These studies in humans found parallelism in preclinical studies documenting that methamphetamine induces microglial activation in the brain (Thomas et al., 2004; Raineri et al., 2012). Along with microglial activation, methamphetamine can increase striatal mRNA expression levels of IL-6 family pro-inflammatory cytokines, leukemia inhibitory factor, oncostatin m, and IL-6 (Robson et al., 2013). These observations are consistent with the idea that the drug might cause neuronal dysfunction via microglia-secreted pro-inflammatory and toxic factors.

In addition to their toxic effects, microglia can alter neuronal excitability by affecting both inhibitory and excitatory synaptic transmission (Sierra et al., 2014). Tremblay et al. (2010) showed that microglia normally contact spines, synaptic terminals, and synaptic clefts in the cortex (Tremblay et al., 2010). Microglia can also regulate basal glutamatergic and GABAergic synaptic transmission in the context of brain injury by a mechanism that involves the increased production of ATP that stimulates the release of brain-derived neurotrophic factor (BDNF) from microglial cells (Tsuda et al., 2003; Davalos et al., 2005). BDNF is a neurotrophin that regulates neuronal survival and differentiation. BDNF also modulates neuronal activity and synaptic plasticity (Santos et al., 2010). Because neurons and microglia express BDNF (Trang et al., 2011), this protein may influence a vast array of functions in the brain. Of specific relationship to our discussion, it has been shown that infusion of BDNF into subcortical structures such as the nucleus accumbens and ventral tegmental area enhances cocaine-induced behavioral sensitization and cocaine seeking (Lu et al., 2004; Graham et al., 2007). In contrast, BDNF infusion into the dorsomedial prefrontal cortex following cocaine self-administration attenuates relapse to cocaine seeking after abstinence; cue- and cocaine prime-induced reinstatement of cocaine-seeking were similarly affected (Whitfield et al., 2011). Some of the effects of cocaine on BDNF appear to be mediated via induced expression of microRNA 212 (Hollander et al., 2010), with the magnitude of BDNF expression being dependent on a homeostatic interaction of microRNA 212 and MeCP2 in the dorsal striatum (Im et al., 2010). Methamphetamine self-administration also causes increased BDNF expression at both mRNA and protein levels (Cadet et al., 2014b). However, since these studies did not clarify the principal sources of BDNF expression, it remains to be determined the extent to which microglial cells might be influencing these drug-induced changes in BDNF expression.

## Conclusions

Addiction of licit and illicit substances can be viewed as maladaptive plastic responses to exposure to agents that impact the expression of various genes and proteins in the brain. Some of these proteins are known to be involved in developmental processes that are dormant during adulthood (Cadet, 2009; Cadet et al., 2014b). Drug-induced elevated expression of some of these proteins could have induced glial proliferation, neuronal dedifferentiation, as well as structural and dysfunctional interactions between glial and neuronal cells (Cadet, 2009). Because glial cells are such an integral part of global neuronal function, it will be very important to develop tool sets that can differentiate the short-term impact of drug-induced dysfunctions of glial cells that might negatively impact long-term brain functions. This is important in view of the fact that many neurodegenerative disorders including Parkinson's disease are thought to be secondary to glia-dependent neuroinflammatory responses (Rogers et al., 2007; Tansey and Goldberg, 2010). These statements implicate a need for novel approaches to the treatment of human addicts that emphasize the development of protective agents that could cause a return of their brains toward baseline homeostasis.

### Conflict of interest statement

The authors declare that the research was conducted in the absence of any commercial or financial relationships that could be construed as a potential conflict of interest.
